# Supplementary figures and images for: Various Stages of Immune Synapse Formation Are Differently Dependent on the Strength of the TCR Stimulus
Source: Int J Mol Sci. 2020 Apr 2;21(7):2475. doi: 10.3390/ijms21072475 (PMC7177831; doi:10.3390/ijms21072475)

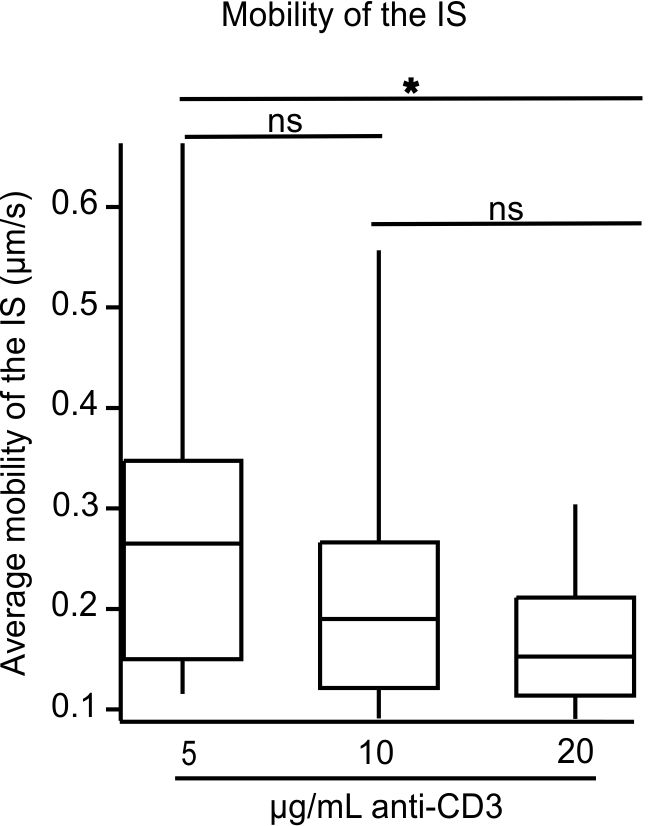

Supplement: Supplementary file 1 [file ijms-21-02475-s001.zip › Figure S1.jpg]

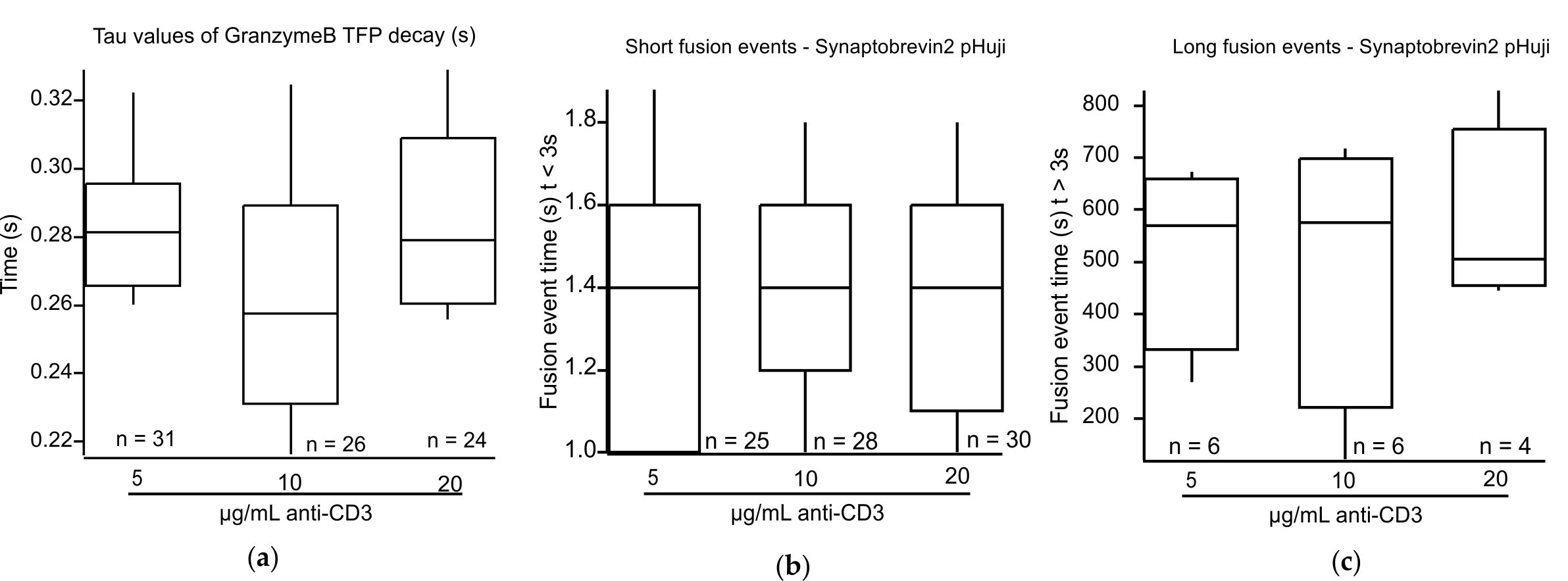

Supplement: Supplementary file 1 [file ijms-21-02475-s001.zip › Figure S2.jpg]
